# Functional analysis of the role of CcpA in *Lactobacillus plantarum* grown on fructooligosaccharides or glucose: a transcriptomic perspective

**DOI:** 10.1186/s12934-018-1050-4

**Published:** 2018-12-28

**Authors:** Yanqing Lu, Sichao Song, Huaixiang Tian, Haiyan Yu, Jianxin Zhao, Chen Chen

**Affiliations:** 1 School of Perfume and Aroma Technology, Shanghai Institute of Technology, Shanghai, 201418 People’s Republic of China; 2 Shanghai-MOST Key Laboratory of Health and Disease Genomics, Chinese National Human Genome Center at Shanghai, Shanghai, 201203 People’s Republic of China; 3 State Key Laboratory of Food Science and Technology, School of Food Science and Technology, Jiangnan University, Wuxi, 214122 Jiangsu Province People’s Republic of China

**Keywords:** *Lactobacillus plantarum*, Catabolite control protein A, Transcriptome, Mixed fermentation, Carbohydrate metabolism, Fatty acid biosynthesis

## Abstract

**Background:**

The catabolite control protein A (CcpA) is a master regulator of many important cellular processes in Gram-positive bacteria. In *Lactobacillus plantarum*, CcpA directly or indirectly controls the transcription of a large number of genes that are involved in carbohydrate metabolism, aerobic and anaerobic growth, stress response and metabolite production, but its role in response to different carbon sources remains unclear.

**Results:**

Here a combined transcriptomic and physiological approach was used to survey the global alterations that occurred during the logarithmic growth phase of wild-type and *ccpA* mutant strains of *L. plantarum* ST-III using fructooligosaccharides (FOS) or glucose as the sole carbon source. The inactivation of *ccpA* significantly affected the growth and production of metabolites under both carbon sources. About 15% of the total genes were significantly altered between wild-type and *ccpA* strains grown on glucose and the value is deceased to 12% when these two strains were compared on FOS, while only 7% were obviously changed due to the loss of CcpA when comparing strains grown on glucose and FOS. Although most of the differentially expressed genes mediated by CcpA are glucose dependent, FOS can also induce carbon catabolite repression (CCR) through the CcpA pathway. Moreover, the inactivation of *ccpA* led to a transformation from homolactic fermentation to mixed fermentation under aerobic conditions. CcpA can control genes directly by binding in the regulatory region of the target genes (mixed fermentation), indirectly through local regulators (fatty acid biosynthesis), or have a double effect via direct and indirect regulation (FOS metabolism).

**Conclusion:**

Overall, our results show that CcpA plays a central role in response to carbon source and availability of *L. plantarum* and provide new insights into the complex and extended regulatory network of lactic acid bacteria.
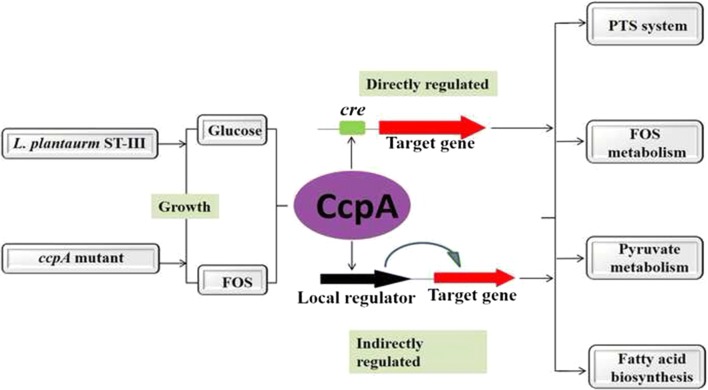

**Electronic supplementary material:**

The online version of this article (10.1186/s12934-018-1050-4) contains supplementary material, which is available to authorized users.

## Background

Carbon catabolite repression (CCR) is one of the most fundamental and highly conserved mechanisms in both Gram-negative and Gram-positive bacteria [1]. This mechanism ensures optimal use of available nutrients and results in a fitness advantage in the natural environment. One of the most important and highly conserved regulators of CCR in low-GC Gram-positive bacteria is catabolite control protein A (CcpA) [2], which is a member of the LacI/GalR family of transcriptional regulators involved in many important cellular processes [3].

In the presence of a preferred carbon source, usually glucose, CcpA is activated by forming a complex with the corepressor Hpr that has been phosphorylated on residue Ser46 (HPr-Ser-P). Hpr can be phosphorylated either at Ser46 or at His15 [4]. In the latter form, it acts in the sugar phosphotransferase system (PTS) for sugar uptake. The CcpA-(Hpr-Ser-P) complex has an increased affinity for particular cis-acting DNA motifs, termed catabolite responsive elements (*cre*), and thereby represses or enhances gene expression, depending on the position of the *cre* in relation to the operator sequence [5]. The consensus sequence of a *cre* site typically has a palindromic nucleotide motif (TGWAANCGNTNWCA, where N = any base; W = A or T), although some variations in nucleotide composition and length have recently been reported [6]. Besides, CcpA also indirectly controls several additional regulators by affecting the expression of these molecules, which in turn alters the transcription of their target genes [5]. Through the interaction, a metabolic network mediated by CcpA is constructed, which plays a major role in the global regulation of metabolism to ensure optimum cell propagation under given growth conditions [7]. However, although CcpA is known to affect expression of ~ 15% of the genomes of numerous bacterial species [8], the contribution of direct versus indirect CcpA-mediated effects on transcription remains unclear.

*Lactobacillus plantarum* is a species of facultative heterofermentative lactic acid bacteria (LAB) found in various environmental niches. Due to its ecological flexibility, *L. plantarum* has developed complex molecular response mechanisms, including CCR mediated by CcpA, to cope with environmental stresses and to survive [9]. Several studies have demonstrated that CcpA directly or indirectly controls the transcription of a large number of *L. plantarum* genes that are involved in carbohydrate metabolism [10, 11], aerobic and anaerobic growth [11], stress response [12] and metabolite production [12]. In our previous studies, we found that fructooligosaccharide (FOS) metabolism in *L. plantarum* is controlled by two gene clusters. Diauxic growth, hierarchical utilization of carbohydrates and repression of FOS-related genes were observed in cultures containing FOS and glucose, suggesting CCR control of FOS utilization [13]. In addition, the inactivation of the *ccpA* gene and verification of the binding of CcpA to the *cre* sites confirmed the dominant role of CcpA in CCR for FOS metabolism. These results demonstrated that the global regulator CcpA controls a large number of important physiological and metabolic processes in *L. plantarum*.

To fully understand the global regulation of CcpA in the carbohydrate metabolism of *L. plantarum*, the role of CcpA in controlling aerobic growth on glucose and FOS was comprehensively investigated via transcriptomic and physiological studies performed on *L. plantarum* ST-III and its *ccpA* mutant strain. Based on these results, we identified a large number of genes controlled by CcpA in different carbon sources, thereby revealing further details of CcpA-mediated regulatory networks in LAB.

## Results

### Growth profiles and metabolite production of the wild-type and *ccpA* mutant strains

**Fig. 1:**
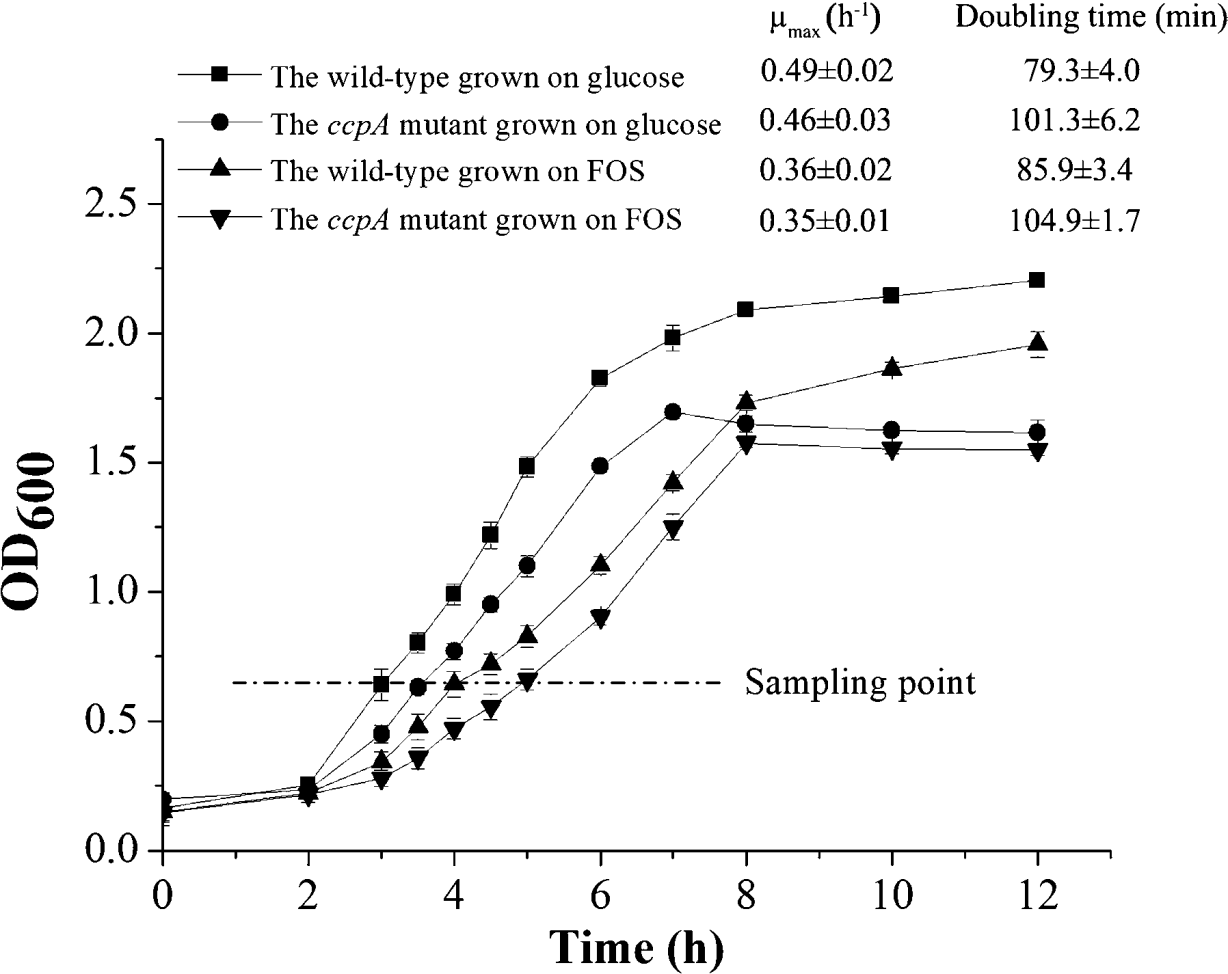
Growth curves of the wild-type and *ccpA* mutant in CDM containing glucose or FOS. Sampling point was chosen for the transcriptomic analysis, the metabolites measurement and RT-qPCR analysis. The μ_max_ for each condition was also calculated and shown in the figure. Data presented are mean values based on two replicate fermentations. Error bars indicate standard deviations

A mutant *ccpA* deletion strain was constructed using the Cre-*lox*-based mutagenesis system [14] to study the regulation of CcpA. *L. plantarum* ST-III and its *ccpA* mutant were cultivated at 37 °C in chemically defined medium (CDM) medium containing glucose or FOS and the growth profiles during fermentation were compared (Fig. 1). The *ccpA* mutant strain exhibited a significantly reduced growth compared with the wild-type strain in glucose (a doubling time of 79.3 ± 4.0 min versus 101.3 ± 6.2 min) and FOS (a doubling time of 85.9 ± 3.4 min versus 104.9 ± 1.7 min). The values of maximal growth rate (μ_max_) were significantly higher for the wild-type than the mutant strain (P-value < 0.05) in the logarithmic phase for both carbon sources. The wild-type strain on glucose exhibited the maximum μ_max_ (0.49 h^−1^ ± 0.02), while the mutant strain on FOS showed the minimum (0.35 h^−1^ ± 0.01). In the stationary phase, the optical density at 600 nm (OD_600_) value of the wild-type grown on glucose and FOS exhibited a slow increase, while the values for the mutant strain showed a weakly declining trend. These data suggest that CcpA severely impairs the growth of *L. plantarum* and the effects are carbohydrate-dependent.

The levels of organic acids generated during fermentation with *L. plantarum* ST-III and its *ccpA* mutant on FOS or on glucose, respectively, are shown in Table 1. The mutant strain grown on glucose produced more acetate and less lactate than the wild-type strain (P-value < 0.05). This situation also occurred in the presence of FOS, although to a lesser extent. In the absence of CcpA, the levels of acetate and lactate did not vary between the strains grown on FOS or glucose. In agreement with the findings of our previous study [15], the levels of formate did not differ among the four conditions.

**Table 1:** Comparison of metabolites resulting from the fermentation at OD_600_ of 0.65 in the four conditions

	Wild-type strain			*ccpA* mutant strain		
	Lactate	Acetate	Formate	Lactate	Acetate	Formate
Glucose	15.4 ± 0.70^Aa1^	3.0 ± 0.21^Cb^	1.3 ± 0.10^Ac^	10.3 ± 0.06^Ca^	7.3 ± 0.12^Ab^	1.5 ± 0.15^Ac^
FOS	12.0 ± 0.60^Ba^	5.6 ± 0.44^Bb^	1.2 ± 0.20^Ac^	10.4 ± 0.32^Ca^	7.2 ± 0.10^Ab^	1.2 ± 0.06^Ac^

Data presented are mean values based on two replicate fermentations. Error bars indicate standard deviations

^1^Values marked with different superscript uppercase letters (ABCD) indicate that those in the same column are significantly different (P-value < 0.05) from other values for the same strains. Values marked with different superscript lowercase letters (abcd) indicate that those in the same row are significantly different (P-value < 0.05) from other values obtained during refrigerated storage

### Profiles of the transcriptome data

To determine the effect of *ccpA* deletion on the transcriptome of *L. plantarum*, the transcriptional profile of the wild-type strain was compared to that of the *ccpA* mutant strain in CDM containing glucose and FOS, respectively. Samples in the early logarithmic phase were chosen for transcriptomic analysis. The statistical data for the transcriptomes of the wild-type and *ccpA* mutant strains are summarized in Additional file 1: Table S1. High-quality paired-end reads were generated for four samples, including 7.0, 5.8, 7.0, and 7.0 million for the wild-type and mutant strains growing on glucose and FOS, respectively (Additional file 1: Table S2). The saturation curves showed that the sequencing became saturated when the number of reads reached 1 million for four samples, and the gene coverage indicated adequate sequencing depth (Additional file 1: Figures S1, S2).

**Fig. 2:**
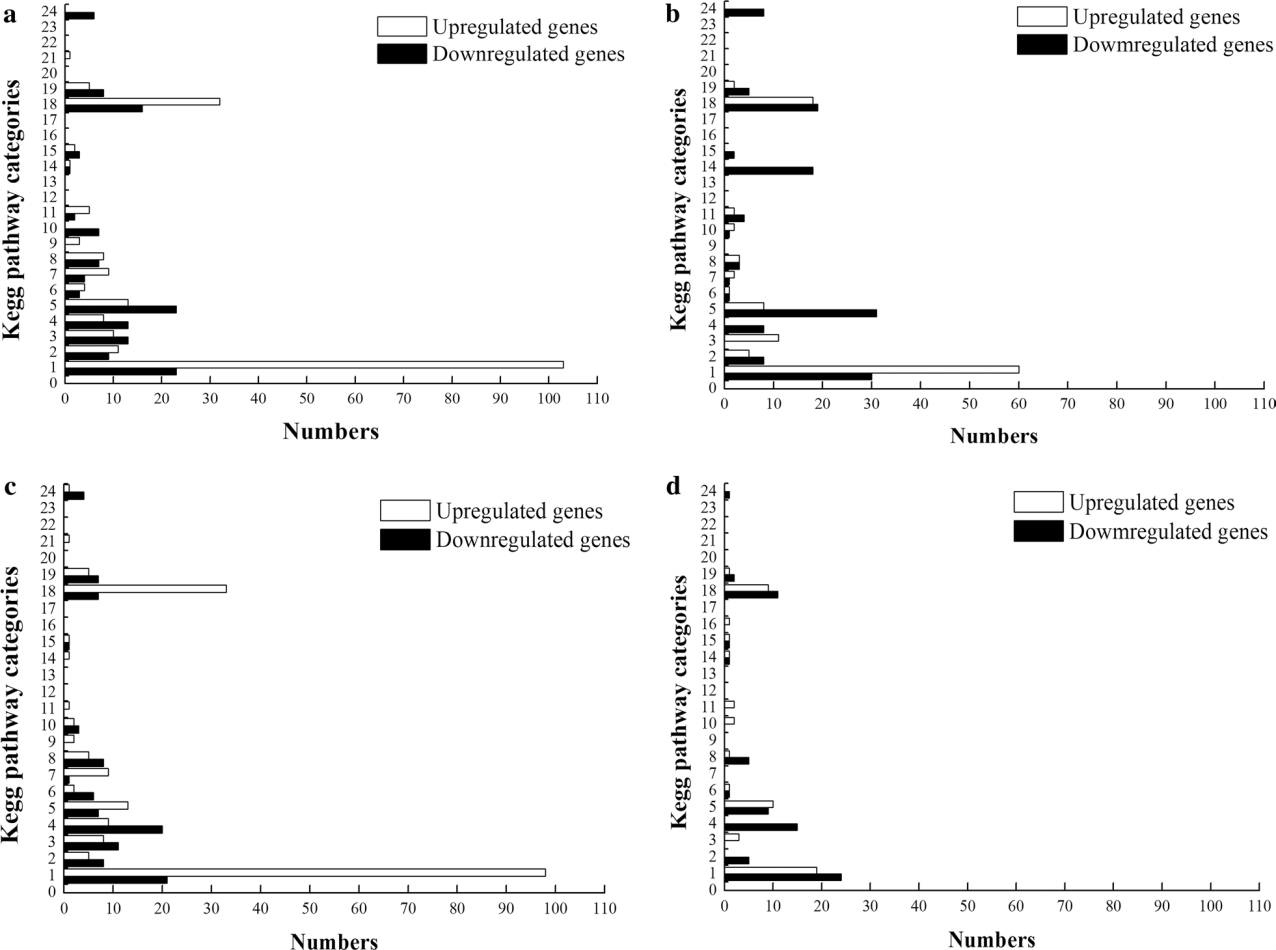
Distribution of upregulated and downregulated genes by KEGG pathway categories of the four pair-wise comparisons. **a** The *ccpA* mutant strain grown on glucose versus the wild-type strain grown on glucose. **b** The *ccpA* mutant strain grown on FOS versus the wild-type strain grown on FOS. **c** The wild-type strain grown on FOS versus the wild-type strain grown on glucose. **d** The *ccpA* mutant strain grown on FOS versus the *ccpA* mutant strain grown on glucose. KEGG pathway categories: 1, carbohydrate metabolism; 2, energy metabolism; 3, lipid metabolism; 4, nucleotide metabolism; 5, amino acid metabolism; 6, metabolism of other amino acids; 7, glycan biosynthesis and metabolism; 8, metabolism of cofactors and vitamins; 9, metabolism of terpenoids and polyketides; 10, biosynthesis of other secondary metabolites; 11, xenobiotic biodegradation and metabolism; 12, enzyme families; 13, transcription; 14, translation; 15, folding, sorting and degradation; 16, replication and repair; 17, RNA family; 18, membrane transport; 19, signal transduction; 20, signaling molecules and interaction; 21, transport and catabolism; 22, cell motility; 23, cell growth and death; 24, Cellular community—prokaryotes

The mean reads per kilo bases per million reads (RPKM) was 419 for the wild-type on glucose, 397 for the mutant on glucose, 414 for the wild-type on FOS, and 408 for the mutant on FOS (Additional file 1: Table S1). To study the gene expression changes of the wild-type and mutant strains during growth on glucose and FOS, respectively, we set our criteria as a fold change > 2 and False discovery rate (FDR) < 0.001 [16] (Additional file 1: Table S1). Two independent factors of the biological processes being studied here were the effects of possession of CcpA and of growth in FOS or glucose, resulting in six pair-wise comparisons. Each pair-wise comparison was visualized as an XY plot of log10 RPKM, with differentially expressed genes marked by different colors (Additional file 1: Figure S3). Among the pair-wise comparisons, the transcriptome profiles of the wild-type in the presence of FOS compared with glucose were analyzed in our previous study [15], and the results are comparable with the current data. To compare and analyze the regulation of CcpA, we focused on three pair-wise comparisons: between wild-type and *ccpA* mutant grown on glucose, between wild-type and *ccpA* mutant grown on FOS, and between *ccpA* mutant cells grown on glucose and FOS. The differentially expressed genes were classified into a wide variety of function categories based on the designations of Kyoto Encyclopedia of Genes and Genomes (KEGG) category (Fig. 2).

To differentiate between direct and indirect CcpA dependent effects, a *cre* consensus motif was built based on the RegPrecise database (http://regprecise.lbl.gov). Using the generated motif to search in the genome of *L. plantarum* ST-III, 72 potential *cre* sites were found. The resulting list of putative *cre* sites was then compared with the genes that were differentially expressed between the wild-type and *ccpA* mutant grown on glucose or FOS. This yielded 63 predicted *cre* sites (Additional file 1: Table S3) that matched the 120 genes differentially expressed in at least one condition. This means that approximately 18% of the differentially expressed genes have a *cre* site and may therefore be directly regulated by CcpA. Notably, the expression of some genes was highly changed, although a *cre* site was not found. CcpA influenced the transcription level of 37 other transcriptional regulators (Additional file 1: Table S4), and thereby may indirectly affect genes that are under the control of these regulators.

To confirm the transcriptome results, 18 key genes identified in the transcriptomic analysis were selected for validation using real-time quantitative PCR (RT-qPCR) (Additional file 1: Table S5). Although the magnitude of gene changes varied between the two methods, the RT-qPCR results displayed similar trends and a high level of correlation with the transcriptome results, implying that the transcriptome data are reliable.

### Comparison of the wild-type and *ccpA* mutant grown on glucose

**Fig. 3:**
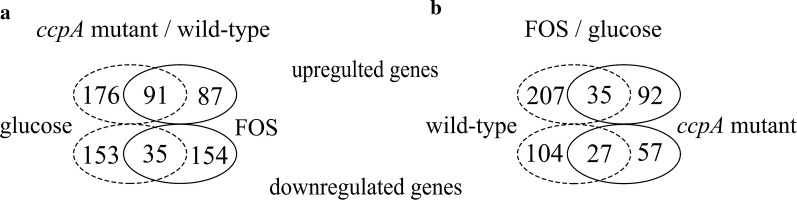
Venn diagrams of the number of genes differentially altered in the four pair-wise comparisons. **a** Number of differentially altered genes in *ccpA* mutant compared to the wild-type, grown on glucose (dotted line) and FOS (full line). **b** Number of differentially altered genes in response to FOS compared with glucose of the wild-type (dotted line) and *ccpA* mutant strains (full line)

**Fig. 4:**
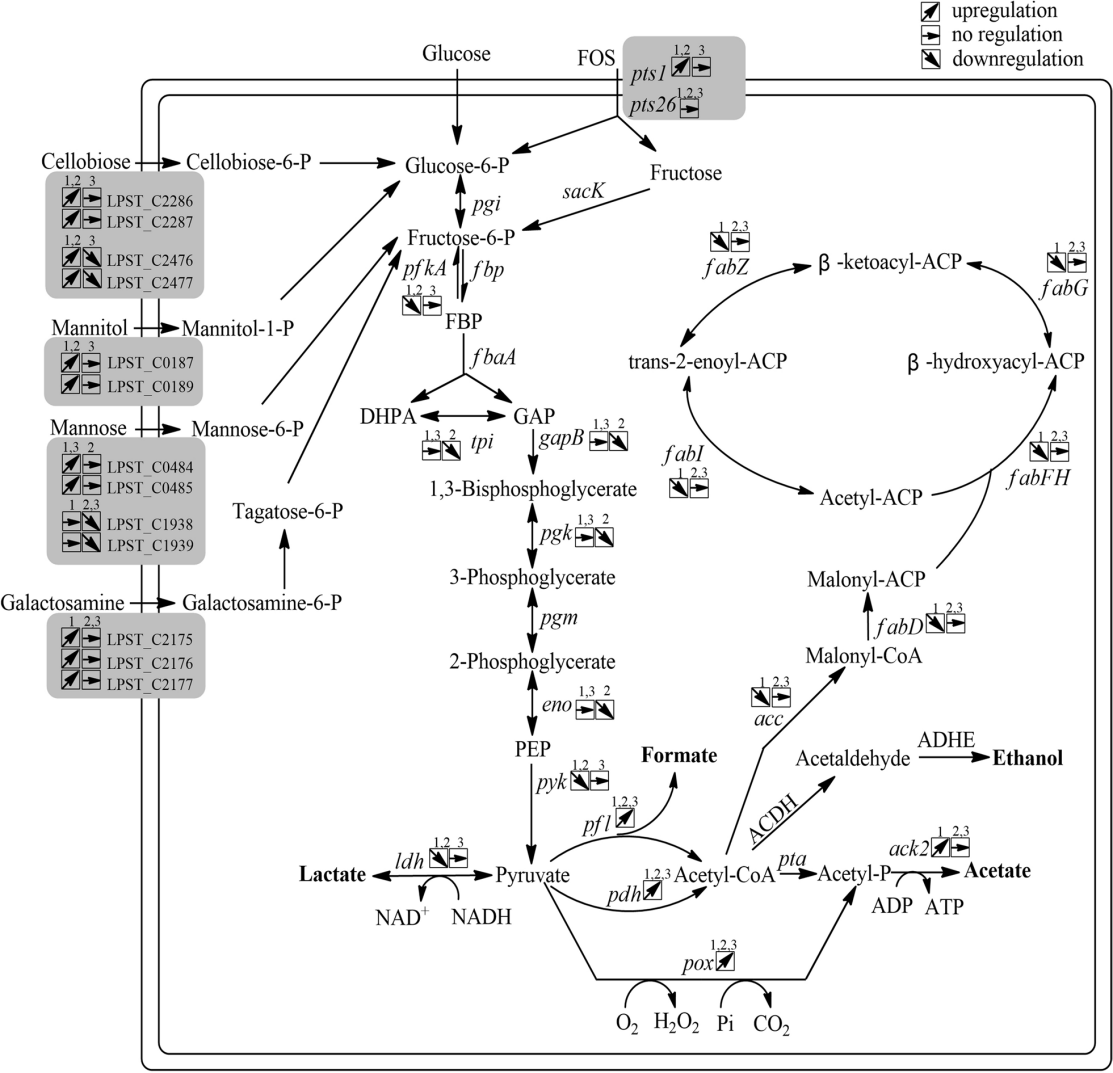
Overview of the key genes and related pathways changed in the transcriptomic analysis. 1, the *ccpA* mutant strain grown on glucose versus the wild-type strain grown on glucose; 2, the *ccpA* mutant strain grown on FOS versus the wild-type strain grown on FOS; 3, the *ccpA* mutant strain grown on FOS verus the *ccpA* mutant strain grown on glucose. Gene annotation downloaded from NCBI: pts, phosphotransferase system; sacK, fructokinase; pgi, glucose-6-phosphate isomerase; fbp, fructose-1,6-bisphosphatase; pfkA, 6-phosphofructokinase; fbpA, fructose-bisphosphate aldolase; tpi, triose-3-phosphate isomerase; gapB, glyceraldehyde-3-phosphate dehydrogenase; pgk, phosphoglycerate kinase; pgm, phosphoglycerate mutase; eno, enolase; pyk, pyruvate kinase; ldh, l-lactate dehydrogenase; pfl, pyruvate formate-lyase; pdh, pyruvate dehydrogenase; pox, pyruvate oxidase; pta, phosphotransacetylase; ack, acetate kinase; acc, acetyl-CoA carboxylase; fabD, ACP-*S*-malonyltransferase; fabF, 3-oxoacyl-ACP synthase II; fabH, 3-oxoacyl-ACP synthase III; fabG, 3-oxoacyl-ACP reductase; fabZ, (3R)-hydroxymyristoyl-ACP dehydratase; fabI, enoyl-ACP reductase

The differentially expressed genes in the wild-type and the mutant strains grown on glucose are listed in Additional file 1: Table S1. Under this condition, 455 genes (15% of all genes) were differentially expressed, which was the largest number of genes changed among the four pair-wise comparisons. Of these, 267 genes were upregulated and 188 were downregulated in the mutant relative to the wild-type strain (Fig. 3a). This demonstrated that the number of genes repressed by glucose in a CcpA-dependent manner greatly exceeded the genes activated by CcpA, which is generally consistent with other analyses for Firmicutes [17–19]. Among those differentially expressed genes, 186 can be grouped into special pathways based on the KEGG database. The largest proportion of upregulated genes is involved in carbohydrate metabolism, including the PTS system, pyruvate metabolism and other processes. The category with the largest proportion of CcpA-induced genes is related to fatty acid biosynthesis (Fig. 4).

The PTS is involved in transporting many sugars into bacteria, including glucose, mannose, fructose and cellobiose [20] and plays a very important role in the utilization of these substrates. Compared with the wild-type strain grown on glucose, seven operons—mannitol-specific transporter (LPST_C0187, LPST_C0189), mannose-specific transporter (LPST_C1938–LPST_C1939), cellobiose-special transporter (LPST_C2286–LPST_C2287, LPST_C2476–LPST_C2477), galactosamine-specific transporter (LPST_C2175–LPST_C2177), glucitol/sorbitol-specific transporter (LPST_C2982–LPST_C2983) and galactitol-specific transporter (LPST_C2896–LPST_C2898)—were upregulated (2.8- to 117.0-fold) by inactivation of *ccpA* (Additional file 1: Table S6). This result clearly indicates that the deletion of *ccpA* activated those genes. It is suggested that in the absence of functional CcpA, HPr is phosphorylated on residue His15 instead of Ser46 [21]. Thus, the HPr-His-P functions as a component of PTS for uptake of different sugars.

Inactivation of *ccpA* also affected pyruvate metabolism in *L. plantarum*. In this comparison, genes encoding the pyruvate dehydrogenase (PDH) complex (LPST_C1775–LPST_C1778), a set of pyruvate formate lyase (PFL) complexes (LPST_C2728–-LPST_C2729), two pyruvate oxidases (POX) (LPST_C2161, LPST_C2933), and an acetate kinase (ACK) (LPST_C0255) were significantly upregulated in the *ccpA* mutant (2.9- to 42.2-fold). The genes encoding l-lactate dehydrogenase (LDH) (LPST_C0295) and pyruvate kinase (PYK) (LPST_C1523–LPST_C1524) were significantly downregulated (Additional file 1: Table S6). The over-expression of POX and ACK and the under-expression of LDH leads to higher acetate production [22, 23] while formate can also form in small amounts due to the over-expression of PFL in certain conditions [17]. All of these findings clearly indicate a shift from homolactic fermentation to mixed fermentation. This transformation is consistent with the metabolite production in comparison between wild-type and *ccpA* mutant strains grown on glucose. *L. plantarum* is a facultative heterofermentative bacterium that can produce acetate and formate at the expense of lactate under certain conditions [15]. The effects of CcpA on the transformation of homolactic fermentation to mixed fermentation have been verified in *L. plantarum* from both metabolic and proteomic perspectives [9, 24].

We also found that some genes involved in glycolysis were significantly altered due to the loss of CcpA. In particular, seven genes (LPST_C0367, LPST_C2283–LPST_C2284, LPST_C2580, LPST_C2877–LPST_C2878, LPST_C2961) encoding 6-phospho-beta-glucosidase (PBG), and the PDH complexes were upregulated (Additional file 1: Table S6). This result suggests that genes involved in glycolysis were affected by the deletion of the *ccpA* gene.

In our previous results, 16 genes in a 10-kbp-region cluster involved in fatty acid biosynthesis were significantly repressed in wild-type cells grown on FOS compared with those grown on glucose [15]. Similarly, all genes in this cluster were downregulated at least 3.3-fold due to the loss of CcpA, except for two genes (LPST_C1341–LPST_C1342) (Additional file 1: Table S6). This demonstrates that those genes are activated in the presence of a functional CcpA and glucose.

### Comparison of the wild-type and its *ccpA* mutant grown on FOS

We next compared the transcriptomes of wild-type and ccpA mutant strains grown in the presence of FOS. The results are listed in Additional file 1: Table S1. A total of 367 genes (12% of all genes) were differentially expressed at least twofold (P-value < 0.05): 178 genes were upregulated and 189 were downregulated in the *ccpA* mutant compared with the wild type (Fig. 3a). Among those differentially expressed genes, 142 can be clustered into the special pathways. Similar to what was observed in glucose-grown cells, carbohydrate metabolism had the most differentially upregulated genes. These genes are mainly involved in the PTS system, pyruvate metabolism, glycolysis, and fructose and mannose metabolism (Fig. 4).

As observed in the transcriptome comparisons described above, some genes encoding PTS components were also induced due to the loss of CcpA in the presence of FOS (2.2- to 12.3-fold increases), including mannitol-specific transporter, cellobiose-specific transporter, and galactitol-specific transporter. This indicates that except for glucose, these genes were also repressed by FOS mediated by CcpA. It is noteworthy that one operon of mannose-specific transporter (LPST_C0484–LPST_C0485) was significantly upregulated in this comparison, while it was repressed due to the loss of CcpA in the presence of glucose. However, another PTS operon (LPST_C1938–LPST_C1939) of this monosaccharide was inversely regulated in this situation (Additional file 1: Table S6). These observations suggest that these genes are regulated by both glucose and FOS, but the mechanism is still unknown.

For pyruvate metabolism, similar to the observation made of cells grown on glucose, several genes exhibited the same alterations in the absence of CcpA, including genes encoding PDH, PFL, POX and LDH. However, the gene encoding ACK did not show a significant difference compared with the wild type (Additional file 1: Table S6). This demonstrate that the transformation of homolactic fermentation to mixed fermentation occur in the presence of FOS, although to a lesser extent. The results of metabolite production confirm this hypothesis.

Some genes involved in glycolysis showed the same alterations under this comparison relative to that of the wild-type and *ccpA* mutant grown on glucose, including some pbg gene complexes (LPST_C0367, LPST_C2283–LPST_C2284, LPST_C2580, LPST_C2877–LPST_C2878, LPST_C2961) and a set of pyk gene complexes (LPST_C1523–LPST_C1524). Notably, the genes (LPST_C0614–LPST_C0617) for enzymes catalyzing the central parts of glycolysis of *L. plantarum*, including glyceraldehyde-3-phosphate dehydrogenase (gap), phosphoglycerate kinase (pgk), triosephosphate isomerase (tpi), and enolase (eno), were downregulated (Additional file 1: Table S6). This alteration was only observed under this comparison, demonstrating that these genes are activated by CcpA in the presence of FOS.

The cluster involved in the fatty acid synthase system did not show significant differences under the FOS condition except for genes (LPST_C1337–LPST_C1341) that were found be upregulated in the mutant strain grown on FOS compared with the wild-type strain (Additional file 1: Table S6). This indicates that the inactivation of *ccpA* has only a minor effect on this cluster in the presence of FOS.

### Comparison of glucose- and FOS-grown cells in the absence of CcpA

A somewhat interesting finding was obtained when the transcriptomes of the *ccpA*-deficient strain growing in FOS and in glucose were compared. Only 211 differentially expressed genes (7% of all genes) (127 upregulated and 84 downregulated in response to FOS compared with glucose) were found under this condition (Fig. 3b). The significantly regulated genes were obviously decreased, compared with the wild-type strain grown on FOS and glucose, suggesting that some of the differentially expressed genes are mainly regulated by CcpA. For example, genes involved in pyruvate metabolism and fatty acid metabolism were differentially expressed only in the presence of a functional CcpA. Among the differentially expressed genes, only 73 can be clustered into the special pathways. Nevertheless, carbohydrate metabolism remained the predominant category (36%) of all differentially expressed genes (Fig. 4).

The differentially expressed genes encoding PTS components were also obviously decreased under this comparison. Notably, an operon (LPST_C1938–LPST_C1939) encoding mannose-specific transporter and the genes (LPST_C2476–LPST_C2477) involved in cellobiose-specific transporter were significantly downregulated in the *ccpA* mutant strain grown on FOS compared with that grown on glucose, while they were upregulated for the wild-type strain in the same comparison (Additional file 1: Table S6).

In our previous study, two gene clusters (*sacPTS1* and *sacPTS26*) were found to be upregulated in the wild-type in response to FOS compared with glucose, proving that these clusters participate in the metabolism of FOS in *L. plantarum* [15]. In the absence of CcpA, the genes for the *sacPTS1* cluster also showed an increase of at least 36.3-fold in the presence of FOS. The *sacPTS26* cluster also tended to exhibit upregulation in transcription with the loss of CcpA, although to a lesser degree (1.5-fold) (Additional file 1: Table S7).

## Discussion

The role of CcpA as a master regulator in Gram-positive bacteria has been revealed in quite a number of regulatory mechanisms of carbon metabolism [8, 12, 25]. CcpA plays such a central role in these regulatory processes that inactivation of the *ccpA* gene leads to partial or complete inhibition of the regulation. We previously found that CcpA-dependent CCR regulates the cellular processes in *L. plantarum*, including carbohydrate metabolism and fatty acid metabolism [15, 26]. Aiming to better understand the regulatory mechanism of CcpA, the global alterations in *L. plantarum* ST-III and its *ccpA* mutant using FOS or glucose as the sole carbon source were revealed via growth profiles, metabolite production and transcriptional analysis.

Inactivation of *ccpA* significantly affected growth and fermentation end-products in *L. plantarum*, in agreement with what has been found for other microorganisms [5, 27]. This confirms CcpA as a master regulator in the control of bacterial metabolism. Similarly, remarkable changes in the transcriptome of *L. plantarum* also occurred with the inactivation of *ccpA*. Among the pair-wise comparisons, the highest differential expression was found in the comparison of the wild-type and *ccpA* mutant strains grown on glucose, while the smallest difference was found in the comparison of glucose- and FOS-grown cells in the absence of CcpA. This result suggests the key role of CcpA in the regulation of carbohydrate metabolism in *L. plantaru*m, especially in a glucose-dependent manner (Fig. 3a). In addition, we found that the loss of CcpA led to the induction of some genes in the presence of FOS (Fig. 3b), including some PTS components, suggesting that CCR can be induced through the CcpA pathway in LAB grown on FOS. This result is consistent with the findings of Abranches et al. [13] that CcpA-dependent CCR is not specific to glucose and several other PTS-transported carbohydrates are also capable of eliciting CcpA-mediated CCR. It seems that the primary trigger for CcpA is sensing of the levels of glycolytic intermediates, such as fructose-1,6-bisphosphate (FBP) [13]. Although FOS is regarded as a poor substrate for triggering CCR, we have demonstrated that FOS is also metabolized by glycolysis in the cytoplasm and FBP is produced [15]. The increase in FBP in cells stimulates the HprK/P-catalyzed formation of P-Ser-HPr, which forms a complex with CcpA and triggers CCR [4]. These results suggest that both glucose and FOS can mediate CcpA-dependent CCR.

CcpA regulates the expression of target genes in a direct or indirect manner [5, 18]. In this study, about 18% of the differentially expressed genes analyzed were presumably subject to direct regulation by CcpA, as indicated by the presence of a *cre* sequence. This value is in good agreement with what has been found for other species [8]. Although the promoter regions of some of the identified genes do not contain *cre* sites, these genes were also regulated in a CcpA-dependent manner, suggesting that CcpA may indirectly affect their expression. Such indirect effects may reflect differences in the generation of metabolites due to *ccpA* inactivation, which might serve as cofactors for the regulation of further genes, and/or the CcpA-dependent control of regulatory proteins [5]. Indeed, CcpA indirectly controlled several additional regulators in the present study (Additional file 1: Table S4), and these regulators might in turn alter the transcription of their target genes.

One interesting outcome of this study concerns pyruvate metabolism. Due to the loss of CcpA, the genes encoding PDH, PFL, ACK, and POX were significantly upregulated, while the LDH complexes was significantly downregulated on both glucose and FOS. As for the measurement of metabolites, a decrease in lactate levels accompanied by the accumulation of acetate was found in the absence of CcpA. These results confirm that the inactivation of *ccpA* leads to a shift from homolactic fermentation to mixed fermentation [17, 24]. It has been shown that CcpA regulates the key genes for mixed fermentation in a direct manner [27]. We also found putative *cre* sites in the promoter regions of the POX and PDH complexes (Additional file 1: Table S3), which support this view. Notably, although the *pfl* gene showed a significant difference from the transcriptome data, the levels of formate did not differ between both strains, as PFL is oxygen sensitive [24].

Fatty acids are essential components of membranes and are important sources of metabolic energy in all organisms [28]. Thus, the fatty acid degradation and biosynthesis pathways must be switched on and off to maintain membrane lipid homeostasis in response to environmental changes. The regulation of these pathways has been studied in a many prokaryote [29]. In our present study, it is shown that beyond controlling carbohydrate metabolism genes, CcpA is also involved in fatty acid metabolism. A reduction in genes involved in fatty acid biosynthesis was induced by *ccpA* inactivation in the presence of glucose. The genes of this 10-kbp region cluster were repressed at least 6.0-fold in response to FOS compared with glucose in the wild-type [15]. Knockout of the *ccpA* gene eliminated this phenomenon, indicating the important role of CcpA in the regulation of fatty acid metabolism. CcpA often indirectly controls local regulators by affecting the fatty acid metabolism in response to environmental changes [28, 30]. For example, Faustoferri et al. [30] demonstrated that CcpA plays an indirect role in the regulation of fatty acid metabolism in *Streptococcus mutans*. CcpA can exert a negative control on the genes encoding the transcriptional regulator FabT, finally affecting the whole gene cluster. A transcriptional regulator (61% identity to FabT) was also identified within the gene cluster in *L. plantarum*, suggesting a similar regulation of fatty acid biosynthesis by the indirect control of CcpA. Though such regulation, *L. plantarum* could maintein membrane lipid homeostasis in response to carbon availability.

Our previous studies demonstrated that the *sacPTS1* and *sacPTS26* clusters involved in FOS metabolism are regulated by CcpA. As expected, with the same carbon source, these two clusters were not significantly altered between the wild-type and mutant strains. However, the two clusters were upregulated in response to FOS compared with glucose due to the loss of CcpA, demonstrating that these two gene clusters may also be affected by other regulators. This deduction is consistent with our previous prediction that two local regulators, SacR1 and SacR2, are involved in the regulation of these clusters. Notably, these local regulators were also activated or repressed by CcpA under certain conditions in this study (Additional file 1: Table S4), suggesting that CcpA mediates the double effect of direct and indirect regulation of FOS metabolism.

## Conclusions

In summary, we performed a whole-transcriptome and metabolic analysis of *L. plantarum* and its *ccpA* mutant grown on glucose and on FOS. Our results show the importance of the global regulator CcpA in *L. plantarum* as a key factor controlling several processes, including carbohydrate metabolism, pyruvate metabolism and fatty acid metabolism. Most genes were affected by CcpA in a glucose-dependent manner; however, FOS also mediated CcpA-dependent CCR. A shift from homolactic fermentation to mixed fermentation occurred in response to *ccpA* inactivation. CcpA appears to mediate the transcriptional response of target genes either directly or indirectly, or has a double effect, and the related mechanism needs further investigation. The data obtained in this study provide new insights into the role of CcpA in the regulation of carbohydrate catabolism by LAB and help to build complex regulatory networks in response to carbon source and availability.

## Methods

### Bacterial strains and growth conditions

*Lactobacillus plantarum* ST-III was kindly provided by Bright Dairy & Food Co., Ltd., China. Construction of the *ccpA* deletion mutants of *L. plantarum* was performed as previously described [26]. *L. plantarum* ST-III and its *ccpA* mutant strain were cultured in de Man-Rogosa-Sharpe (MRS) broth (Merck, Darmstadt, Germany) at 37 °C without agitation for routine manipulations. For transcriptomic and physiological analysis, cells were grown in CDM supplemented with either FOS or glucose as the carbon sources, which were sterilized separately through a sterile 0.2-mm filter and added to the fermentation medium. The FOS used in this study was a commercial compound supplied by Meiji Seika Kaisha (Tokyo, Japan), comprising 37.3% (w/w) 1-kestose, 49.1% (w/w) nystose, 9.8% (w/w) fructosyl-nystose, 2.3% (w/w) sucrose, and 1.3% (w/w) glucose and fructose. The monosaccharides and disaccharides in FOS have been proven to have negligible effects on the growth of *L. plantarum* and do not affect the subsequent analysis [15].

### Fermentation and sampling

*Lactobacillus plantarum* ST-III and its *ccpA* mutant strains were propagated in parallel for two passes in CDM with 1% (w/v) FOS or glucose as the sole carbon source, respectively. Cultures of each sugar were then transferred with 2% (v/v) inoculum into 500 mL of CDM containing the same sugar. The cells were grown under aerobic conditions without agitation at 37 °C using a bioreactor (Bioflo model 115, New Brunswick Scientific Co., Edison, NJ, USA) by flushing with sterile air (0.1 v/v min). Growth was monitored by measuring OD_600_. When OD_600_ values reached 0.65, the cultures grown on FOS and glucose were harvested by centrifugation (8000×*g*, 10 min, 4 °C), and the cell pellets and supernatants were collected, respectively. The cell pellets were flash frozen for storage at − 80 °C for further RNA isolation. The supernatant was filtered through a 0.45-µm nylon filter and the production of lactate, acetate and formate was analyzed by high-performance liquid chromatography as previous described [15]. Two replicate fermentations were carried out for each treatment.

### RNA isolation and transcriptome analysis

Total RNA was isolated using TRIzol reagent (Invitrogen, Carlsbad, CA, USA) following the standard protocol. The final total RNA was dissolved in 200 μL RNase-free water. The concentration of total RNA was determined by NanoDrop (Thermo Scientific, USA), and the RNA integrity value was checked using the RNA 6000 Pico LabChip of an Agilent 2100 Bioanalyzer (Agilent, Palo Alto, CA, USA).

Total RNA was then incubated with 10 U DNase I at 37 °C for 1 h, then ribosomal RNA was depleted using Ribo-Zero rRNA Removal Kit Bacteria (Illumina, San Diego, CA, USA). For the transcriptomic analysis, mRNA was fragmented and strand-specific cDNA libraries were constructed using KAPA Stranded RNA-Seq Kit (Kapa Biosystems, Boston, MA, USA) according to the manufacturer’s instructions. The 2 × 150 bp paired-end sequencing was performed on an Illumina X10 (Illumina, Inc., San Diego, CA, USA).

### Bioinformatics analysis

The reads of each sample were mapped to the reference genome of *L. plantarum* ST-III (accession number: CP002222.1) using bowtie with a default parameter [31]. The read number of each gene was first transformed into RPKM [32], then differently expressed genes were identified by DEGseq package using the MA-plot-based method with random sampling (MARS) [33]. FDR was used to determine the threshold of the P-value for this analysis. “FDR < 0.001 and absolute value of fold change > 2” was used as the threshold to judge the significance of contig expression differences in the different sets of comparisons.

The enrichment of KEGG pathways and classification for a given gene list was calculated using a classical hypergeometric distribution statistical comparison of a query gene list against a reference gene list. The calculated P-values underwent FDR correction, with corrected P-value < 0.05 as the threshold. KEGG pathways fulfilling this condition were defined as significantly enriched pathways in regulated genes. The transcriptomic data have been deposited in the BioProject at the National Center for Biotechnology Information (NCBI) with the accession number PRJNA493968.

A *cre* site prediction was performed by scanning all upstream regions in the genome of *L. plantarum* ST-III based on the positional frequency matrix that was constructed in our previous studies [26]. The scores of candidate sites were defined as the sum of positional nucleotide weights as previously described [34]. The resulting predictions of putative *cre* sites were compared with the genes that were differentially expressed in the mutant strain relative to the wild-type strain under both carbon sources.

### Confirmation of transcriptomic results by real-time quantitative PCR

Total RNA was isolated as described above and was treated with DNase I. RT-qPCR was performed on RNA samples to confirm the absence of DNA contamination using 16S primers. The cDNA synthesis was performed with a PrimeScript RT reagent kit (Takara, Dalian, China), according to the manufacturer’s instructions. The primers used in RT-qPCR are listed in Additional file 1: Table S8. RT-qPCR was carried out using Power SYBR Green PCR Master Mix (Applied Biosystems, Foster City, CA, USA) with a 7300 fast real-time PCR system (Applied Biosystems). Gene expression was normalized by the 2^−ΔΔCt^ method and the 16S rRNA gene was used as the normalized standard. All of the samples were measured in triplicate.

### Statistical analysis

Statistical analyses were conducted using SPSS (v. 19.0, IBM SPSS, Chicago, Ill., USA). Student’s t-test was used to determine statistical differences. Differences between samples with a P-value < 0.05 were considered statistically significant.

## Additional file

**Additional file 1: Figure S1.** The sequencing saturation analysis of transcriptome data in the four conditions. Two replicate fermentations were carried out for each treatment and a total of 8 saturation curves were generated. **Figure S2.** Gene coverage distribution of transcriptome data in the four conditions. The percentage means the ratio of numbers of genes in different coverage intervals to the total number of genes. **Figure S3.** Representative volcano plots of six pair-wise comparisons. **Table S1.** Overview of whole transcriptome data in this study as determined by the RNA-seq analysis. **Table S2.** Summary of transcriptome data for wild-type and *ccpA* mutant strain grown on different carbon sources. **Table S3.** List of genes with putative *cre* sites that were significantly affected in the transcriptome analysis. **Table S4.** Local regulators subject to the regulation by CcpA. **Table S5.** Validation of transcriptome data by RT-qPCR on 18 selected genes. **Table S6.** Gene expression profiles of the key pathways in four pair-wise comparisons. **Table S7.** The gene expression of *sacPTS1* and *sacPTS26* gene clusters in four pair-wise comparisons. **Table S8.** The primers for RT-qPCR analysis with target gene information.

## Abbreviations

CcpA: catabolite control protein A
FOS: fructooligosaccharides
CCR: carbon catabolite repression
PTS: phosphotransferase system
*cre*: catabolite responsive elements
LAB: lactic acid bacteria
MRS: De Man-Rogosa-Sharpe
CDM: chemically defined medium
OD: optical density
RPKM: reads per kilo bases per million reads
MARS: MA-plot-based method with random sampling
FDR: false discovery rate
KEGG: kyoto encyclopedia of genes and genomes
NCBI: national center for biotechnology information
RT-qPCR: real-time quantitative PCR
PDH: pyruvate dehydrogenase
PFL: pyruvate formate lyase
POX: pyruvate oxidases
ACK: acetate kinase
LDH: lactate dehydrogenase
PYK: pyruvate kinase
PBG: phospho-beta-glucosidase
GAP: glyceraldehyde-3-phosphate dehydrogenase
PGK: phosphoglycerate kinase
TPI: triosephosphate isomerase
ENO: enolase
FBP: fructose-1,6-bisphosphate
