# Supplementary figures and images for: Functional analysis of the role of CcpA in Lactobacillus plantarum grown on fructooligosaccharides or glucose: a transcriptomic perspective
Source: Microb Cell Fact. 2018 Dec 28;17:201. doi: 10.1186/s12934-018-1050-4 (PMC6309078; doi:10.1186/s12934-018-1050-4)

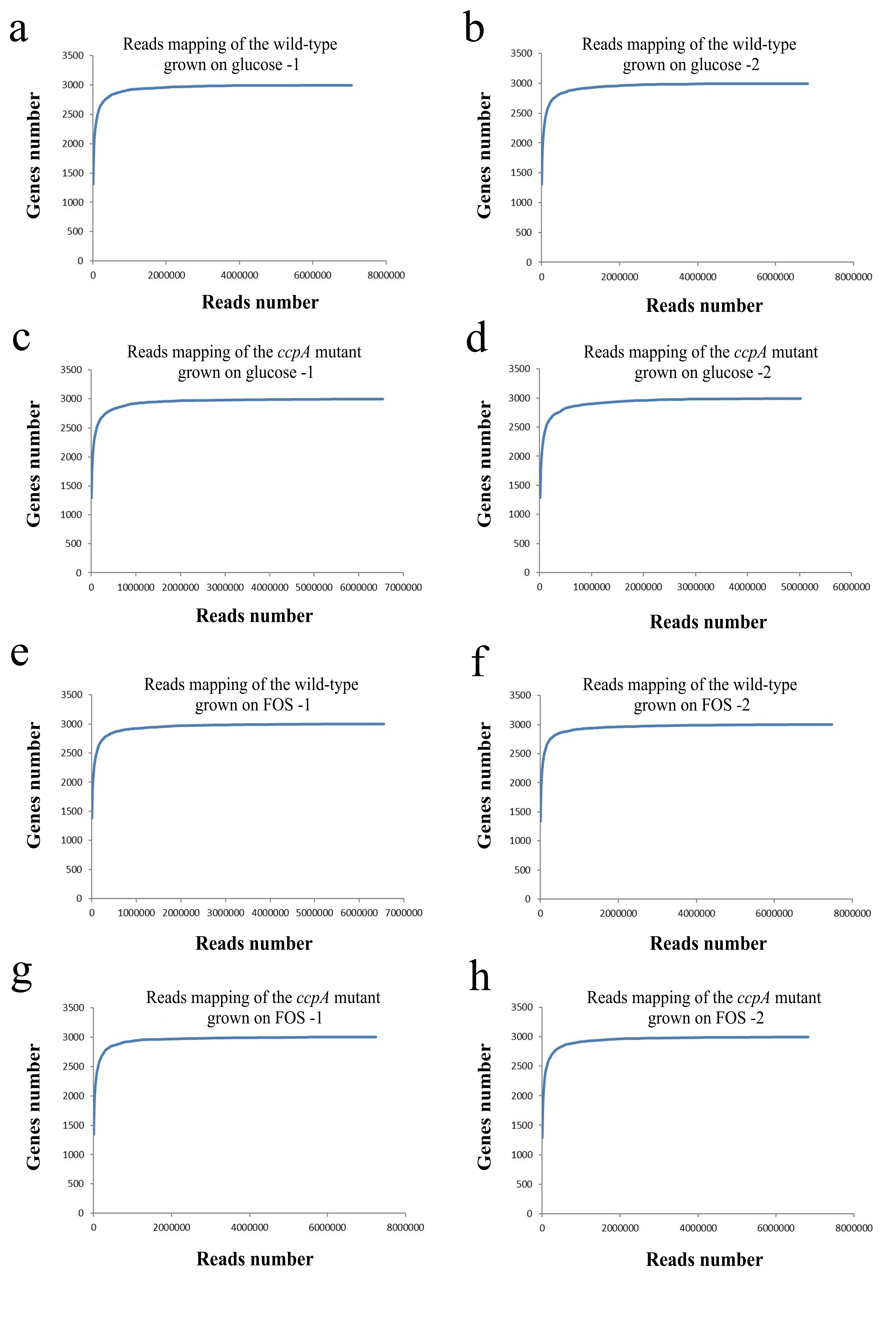

Supplement: Supplementary file 1 — Additional file 1: Figure S1. The sequencing saturation analysis of transcriptome data in the four conditions. Two replicate fermentations were carried out for each treatment and a total of 8 saturation curves were generated. Figure S2. Gene coverage distribution of transcriptome data in the four conditions. The percentage means the ratio of numbers of genes in different coverage intervals to the total number of genes. Figure S3. Representative volcano plots of six pair-wise comparisons. Table S1. Overview of whole transcriptome data in this study as determined by the RNA-seq analysis. Table S2. Summary of transcriptome data for wild-type and ccpA mutant strain grown on different carbon sources. Table S3. List of genes with putative cre sites that were significantly affected in the transcriptome analysis. Table S4. Local regulators subject to the regulation by CcpA. Table S5. Validation of transcriptome data by RT-qPCR on 18 selected genes. Table S6. Gene expression profiles of the key pathways in four pair-wise comparisons. Table S7. The gene expression of sacPTS1 and sacPTS26 gene clusters in four pair-wise comparisons. Table S8. The primers for RT-qPCR analysis with target gene information. [file 12934_2018_1050_MOESM1_ESM.zip › Figure. S1.jpg]

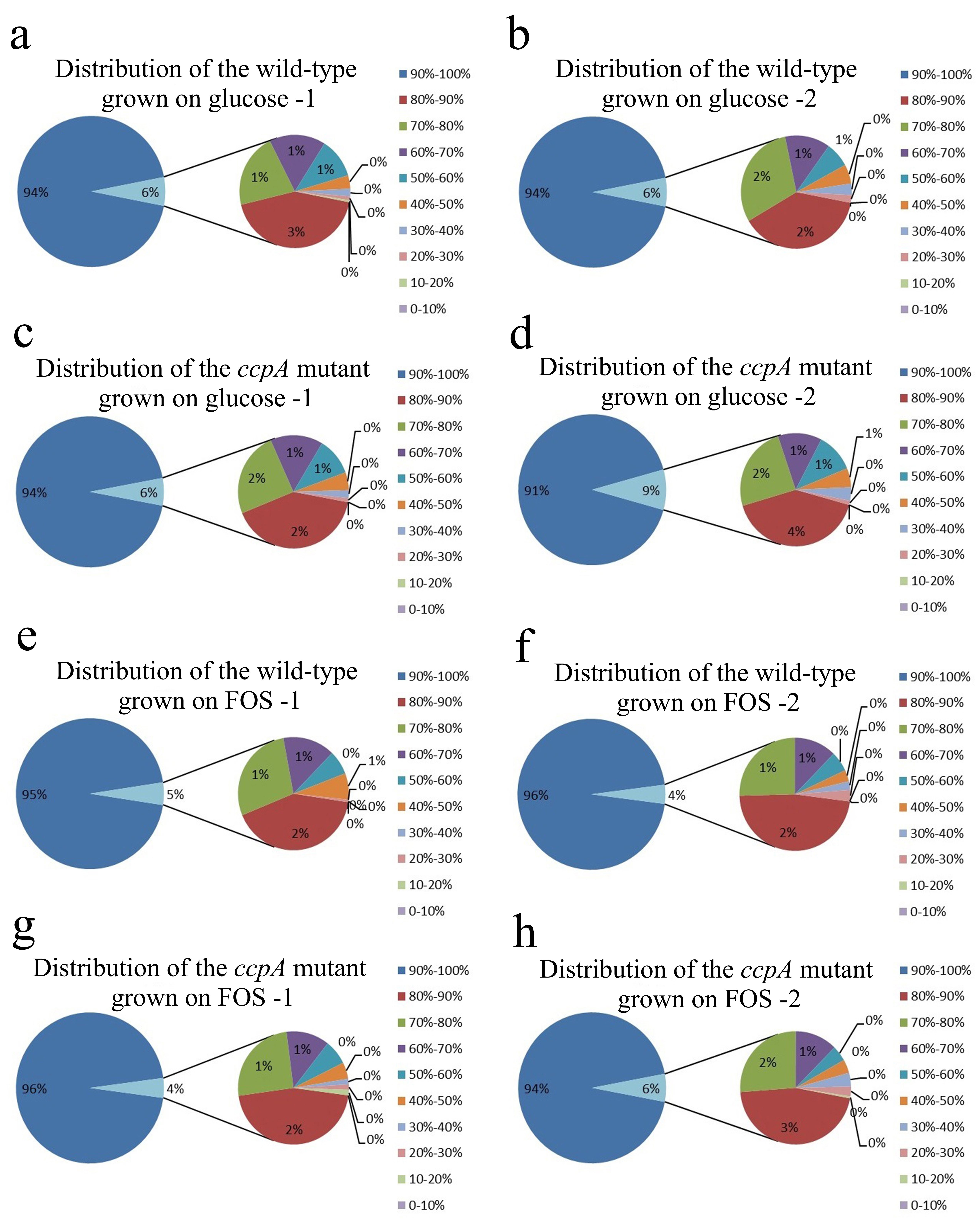

Supplement: Supplementary file 1 — Additional file 1: Figure S1. The sequencing saturation analysis of transcriptome data in the four conditions. Two replicate fermentations were carried out for each treatment and a total of 8 saturation curves were generated. Figure S2. Gene coverage distribution of transcriptome data in the four conditions. The percentage means the ratio of numbers of genes in different coverage intervals to the total number of genes. Figure S3. Representative volcano plots of six pair-wise comparisons. Table S1. Overview of whole transcriptome data in this study as determined by the RNA-seq analysis. Table S2. Summary of transcriptome data for wild-type and ccpA mutant strain grown on different carbon sources. Table S3. List of genes with putative cre sites that were significantly affected in the transcriptome analysis. Table S4. Local regulators subject to the regulation by CcpA. Table S5. Validation of transcriptome data by RT-qPCR on 18 selected genes. Table S6. Gene expression profiles of the key pathways in four pair-wise comparisons. Table S7. The gene expression of sacPTS1 and sacPTS26 gene clusters in four pair-wise comparisons. Table S8. The primers for RT-qPCR analysis with target gene information. [file 12934_2018_1050_MOESM1_ESM.zip › Figure. S2.jpg]

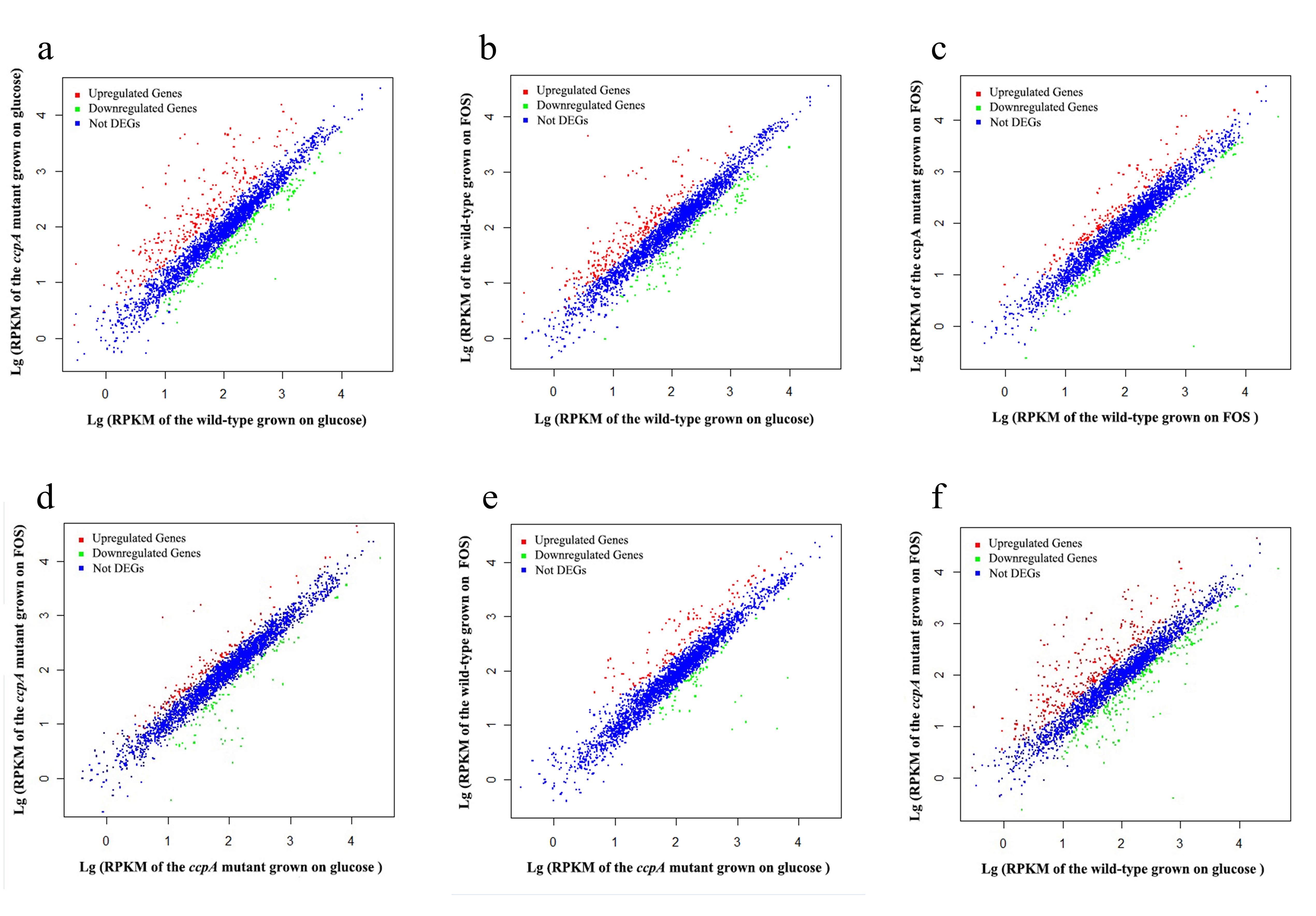

Supplement: Supplementary file 1 — Additional file 1: Figure S1. The sequencing saturation analysis of transcriptome data in the four conditions. Two replicate fermentations were carried out for each treatment and a total of 8 saturation curves were generated. Figure S2. Gene coverage distribution of transcriptome data in the four conditions. The percentage means the ratio of numbers of genes in different coverage intervals to the total number of genes. Figure S3. Representative volcano plots of six pair-wise comparisons. Table S1. Overview of whole transcriptome data in this study as determined by the RNA-seq analysis. Table S2. Summary of transcriptome data for wild-type and ccpA mutant strain grown on different carbon sources. Table S3. List of genes with putative cre sites that were significantly affected in the transcriptome analysis. Table S4. Local regulators subject to the regulation by CcpA. Table S5. Validation of transcriptome data by RT-qPCR on 18 selected genes. Table S6. Gene expression profiles of the key pathways in four pair-wise comparisons. Table S7. The gene expression of sacPTS1 and sacPTS26 gene clusters in four pair-wise comparisons. Table S8. The primers for RT-qPCR analysis with target gene information. [file 12934_2018_1050_MOESM1_ESM.zip › Figure. S3.jpg]
